# Memristive Devices Based on Two-Dimensional Transition Metal Chalcogenides for Neuromorphic Computing

**DOI:** 10.1007/s40820-021-00784-3

**Published:** 2022-02-05

**Authors:** Ki Chang Kwon, Ji Hyun Baek, Kootak Hong, Soo Young Kim, Ho Won Jang

**Affiliations:** 1 Department of Materials Science and Engineering, Research Institute of Advanced Materials, Seoul National University, Seoul, 08826 Republic of Korea; 2 Interdisciplinary Materials Measurement Institute, Korea Research Institute of Standards and Science (KRISS), Daejeon, 34133 Republic of Korea; 3 Department of Materials Science and Engineering, Institute of Green Manufacturing Technology, Korea University, Seoul, 02841 Republic of Korea; 4 Advanced Institute of Convergence Technology, Seoul National University, Suwon, 16229 Korea

**Keywords:** Two-dimensional materials, Memristors, Neuromorphic computing, Artificial synapses, Transition metal chalcogenides

## Abstract

Two-dimensional (2D) transition metal chalcogenides (TMC) and their heterostructures are appealing as building blocks in a wide range of electronic and optoelectronic devices, particularly futuristic memristive and synaptic devices for brain-inspired neuromorphic computing systems. The distinct properties such as high durability, electrical and optical tunability, clean surface, flexibility, and LEGO-staking capability enable simple fabrication with high integration density, energy-efficient operation, and high scalability. This review provides a thorough examination of high-performance memristors based on 2D TMCs for neuromorphic computing applications, including the promise of 2D TMC materials and heterostructures, as well as the state-of-the-art demonstration of memristive devices. The challenges and future prospects for the development of these emerging materials and devices are also discussed. The purpose of this review is to provide an outlook on the fabrication and characterization of neuromorphic memristors based on 2D TMCs.

## Highlights

- Based on the benefits of two-dimensional (2D) transition metal chalcogenides (TMC) materials, the operating concepts and basics of memristors for neuromorphic computing are introduced.
- The prospects of 2D TMC materials and heterostructures are reviewed, as well as the state-of-the-art demonstration of 2D TMCs-based memristors for neuromorphic computing applications.
- The most recent advances, current challenges, and future prospects for the manufacture and characterization of memristive neuromorphic devices based on 2D TMCs are discussed.

## Graphical abstract

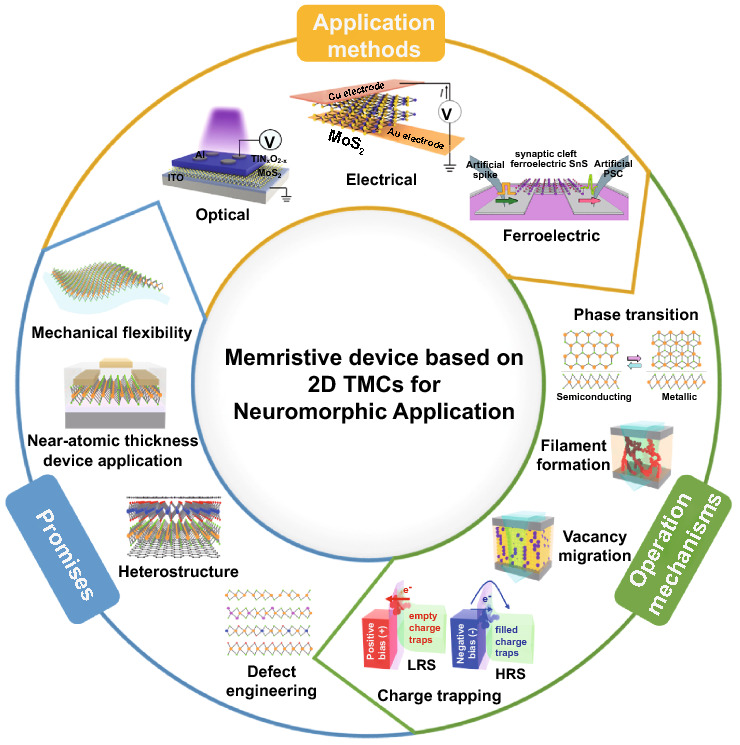

## Introduction

Von Neumann proposed a computational architecture characterized by separation and linear interaction between the memory and central processing unit after the development of metal–oxide–semiconductor (MOS) field-effect transistors (FETs) as key devices of integrated circuits [1, 2]. Because of this systemic configuration, traditional computing devices face inefficiencies resulting from slow data transfer and high power consumption, which is insufficient for big data using artificial neural networks [3–5]. Because of this inefficiency and high power consumption, extensive research has been conducted into neural network architectures inspired by human brains [6]. The distinct architecture and functionality of the human brain allow for the processing of a large amount of information with extremely low power consumption (20 W), mimicking the neural network system composed of neurons and synapses [7]. In recent decades, artificial neural networks (ANNs) have been proposed to solve von Neumann’s bottleneck from traditional computing architectures inspired by the human brain, which has highly parallel computing operation and adaptive learning. There are three critical aspects to mimicking the behavior of biological synapses: (1) conductance states, (2) conductance ratio, and (3) spike-timing-dependent signals [8–10]. The synaptic weight updates perform the learning and extraction process, and the hundreds of conductance states allow for improved learning capability. The minimum and maximum conductance ratio (on/off) of the synapse is translated to the dynamic range, which must be demonstrated. To achieve parallel operation, low power consumption, and lower device volume on spiking neural networks, the development of time-dependent electrical or biological stimuli transmission between neurons is critical [11–13].

A variety of device architectures, including complementary MOS (CMOS) transistors, ferroelectric and gate-tunable transistors, and memristors, have been used to mimic biological synapses using electronic and optoelectronic devices [14–17]. Because of their hysteric I–V characteristics with nonvolatile resistive switching behavior, the memristors (combination of memory and resistor) proposed by Chua et al. [18, 19] have been regarded as one of the most promising candidates in neuromorphic computing systems. These devices can also be used in a large-scale crossbar array architecture with top and bottom electrode lines. Because of these benefits, remarkable progress has been made in developing memristor-based neuromorphic devices to mimic essential synaptic functions such as short-term plasticity (STP), long-term plasticity (LTP), short-term depression (STD), long-term depression (LTD), paired-pulse facilitation (PPF), and spike-time(-voltage and -rate)-dependent plasticity (STDP, SVDP, and SRDP) [20–22].

STP, STD, LTP, and LTD are all linked to potentiation and depression, which are important synaptic weight parameters. STP is defined as the temporal increase of synaptic weight followed by a rapid decrease back to its original state. LTP is the permanent change in synaptic weight caused by a series of external stimuli. When the negative pulse is delivered, the temporal decay of synaptic weight is displayed, indicating an STD state. In the case of consecutive pulses, however, a permanent low value of synaptic weight will be updated (LTD). Another type of short-term plasticity, PPF, is characterized by an increase in synaptic weight in sequentially evoked postsynaptic potentials. Furthermore, the STDP, verifying the Hebbian learning rule, and the SRDP, determining the polarity of synaptic weight by spike-timing and spike firing rate, provide the important information related to synaptic connectivity upon pre- and-postsynaptic terminals [23–25]. A breakthrough in the development of new materials and the design of novel device architectures is required to achieve these functionalities with low power consumption and multi-level resistance control with high reliability.

As a new building block for developing memristive and synaptic devices, two-dimensional (2D) near-atom-thickness materials have attracted the interest of researchers. The atomically thin 2D materials, which could be used to create futuristic artificial synaptic devices, exhibit unique electronic and mechanical properties such as interfacial charge trapping, ferroelectric/ferromagnetic, polymorphism, and phase transformation [26, 27]. Among these 2D materials, 2D transition metal chalcogenides (TMCs) in particular have multiple polymorphs as well as a crystal structure and favorable phase states. There are several operation mechanisms in 2D TMC-based memristive and synaptic devices, including interfacial electrostatic modulations, charge transfer, phase transformation, and gate-tunable energy band shift, and so on. Furthermore, since the 2D TMC layers can be used as LEGO-like building blocks to create a variety of heterostructures, new possibilities for research into various types of electronic/optoelectronic memristors and synaptic devices have been opened. By stacking different 2D materials, a variety of vdW heterostructures with richer optoelectronic properties can be created, allowing for the realization of retinomorphic sensors which is integrated optical perception, memory, and computation functions in crossbar array devices [28]. Under external optical/electrical stimuli, changes in conductivity of 2D materials and their heterostructures enable perception of optical information and conversion of that information into electrical signals for transmission and processing, similar to retina [29, 30]. Integration of optical sensors with memristive/neuromorphic devices based on 2D TMC heterostructures is difficult to demonstrate artificial vision and motion detection and recognition. The specific promises of 2D materials and 2D TMCs will be discussed in further detail.

In this review, we present the recent development of memristive and neuromorphic device applications based on 2D TMCs. We begin with a brief overview of synaptic devices and their synaptic function for memory storage. Following a discussion of the remarkable properties and operation mechanisms of 2D TMCs, we will discuss recent advances in high-performance 2D TMC-based memristors and neuromorphic devices. Finally, we provide a brief overview of the current challenges and prospects for the development of 2D TMCs and their applications in devices.

## Emergence of 2D TMC-Based Memristive and Neuromorphic Devices

### Artificial Synapses and Synaptic Plasticity

In the nervous system of the human brain, a synapse is defined as a junction between two neurons that serves as an essential path for transmitting nervous impulses from presynaptic neurons to postsynaptic neurons. An electrical spike, called an action potential, is generated when an electrical signal is sent to a presynaptic neuron. Then, it triggers the impulse in the postsynaptic neuron by releasing chemicals, so-called neurotransmitters, through the synaptic cleft [31]. Artificial synapses imitate biological synapses in neuromorphic computing systems to realize brain-like massively parallel and fault-tolerant computation in non-von-Neumann computing. Hardware implementation of the artificial synaptic devices begins with a structure consisting of top and bottom electrodes corresponding to the presynaptic and the postsynaptic neuron, respectively, and a memristive material that emulates synaptic signals [32, 33].

The basic requirements for artificial synapse are analog resistance change, synaptic weight regulation, and practicable synaptic plasticities [34–36]. In the artificial synapse, the resistance states of the devices constitute the synaptic weight that refers to the strength or amplitude of the connection between the presynaptic and the postsynaptic neurons, corresponding to the amount of effect that firing one neuron has on another in the biological synapse. Therefore, the resistance value of the device should be gradually changeable, which means the multi-state operation must be capable [37]. There are two directions for synaptic weight regulation: potentiation and depression. The potentiation indicates an increase in synaptic weight (increased conductance), and depression indicates a decrease in synaptic weight (decreased conductance). There are four types of potentiation and depression: STP, STD, LTP, and LTD [38]. STP is accomplished via the temporal improvement of a synaptic weight in response to relatively incompact input pulses and then rapidly decays to its initial activity state. On the contrary, a dense input train with shorter pulse intervals attains a permanent enhancement in synaptic strength, called LTP. With regard to depression in synaptic weight, the temporal decline in synaptic weight represents an STD state, but a permanent degradation of synaptic connection determines an LTD state. This modification of synaptic weight facilitates information storage and processing in the neuromorphic system.

Synaptic plasticity stands for the activity-dependent adjustment in the strength or effectiveness of synaptic transmission over time at the original state [39, 40]. In 1949, Hebbian ascertained that when both presynaptic and postsynaptic neurons fired simultaneously, the synaptic weight between the connected neurons changed, which could be the “first law” of synaptic plasticity [41, 42]. STDP, one of the cardinal forms of synaptic plasticity, is a temporally asymmetric configuration of Hebbian learning provoked by close correlations of spiking time between pre- and postsynaptic neurons. With STDP, the synaptic weight variance, Δ*w*, is expressed as a function of spiking time difference between pre- and postsynaptic neurons, Δ*t*, where positive Δ*t* represents the arrival of presynaptic spike before postsynaptic spike and negative Δ*t* is the opposite. In the case of positive Δ*t* with a small value, which means the presynaptic spike reaches slightly before that of postsynaptic, it generally leads to LTP at many synaptic types. In contrast, the negative Δ*t* with a small absolute value results in LTD in identical synapses. In either case, the smaller the absolute value of Δ*t*, the larger the absolute value of Δ*w*. Therefore, if Δ*t* is positive, the synaptic weight is strengthened as Δ*t* decreases and vice versa. The immediate response of the STDP function to the relative spiking time proposes the potential of time-dependent coding schemes at the level of msec time scale in neuromorphic computing [25].

**Fig. 1:**
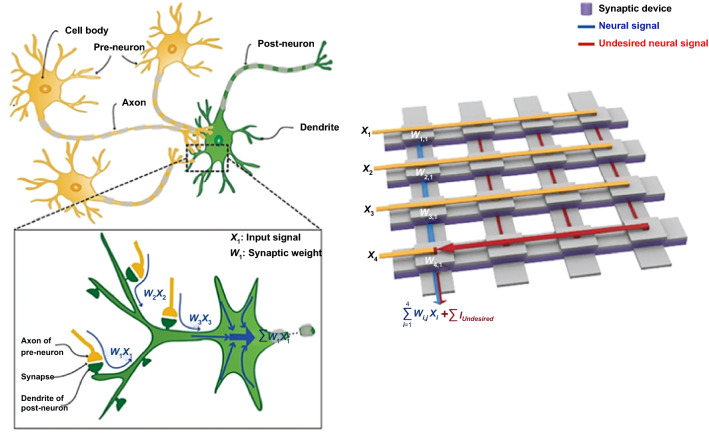
Illustration of the similarities between biological and memristor-based artificial synapses and their neural networks. Reproduced with permission from Ref. [46]. Copyright 2018, IEEE Nanotechnology Magazine. (Color figure online)

Neural networks that mimic the way of signal processing in biological neurons operate as massive parallel vector–matrix multiplication algorithms for learning. The matrices consist of a two-dimensional array of contacts between the perpendicularly crossed top and bottom electrodes arranged in an *x*–*y* format as shown in Fig. 1 [43]. Furthermore, the learning algorithms are operated by the contacts of the matrix located on each column intersection with the specific synaptic weights pre-trained in crossbar arrays [44, 45]. Therefore, for the hardware implementation of the neural network, it is essential to demonstrate the crossbar array structure of neuromorphic devices. The voltage pulses are delivered to the pre-synaptic (top electrode) side. The current flow was produced by external stimuli through the networks, and the charges are collected and integrated at the postsynaptic (bottom electrode) side. The synaptic weight updates can be modulated by controlling spike-timing from synaptic events which are STDP rules [43, 46]. Furthermore, through the crossbar array architecture demonstration, the CMOS-based current memory technology can be emulated to develop the next-generation neuromorphic computing devices. Recently, there are many attempts to develop the crossbar array structures using various memristor-based devices. Among them, Li et al*.* emulated memory behaviors and pattern learning in a 3 × 3 crossbar array with vertical Au/CuInP_2_S_6_ (CIPS)/Ti memristors [47]. Pattern learning is demonstrated by programming the synaptic weights of each of the nine CIPS devices in the array into a potentiation or depression state, respectively, corresponding to the input pattern. Thus, the simplified “s,” “n,” and “u” letters can be encoded and stored as 3 × 3 pixel images based on a crossbar array using the high- and low-resistance states of the devices.

### Promise of 2D TMCs

#### Advantages of 2D Materials in Neuromorphic Devices

There are two different properties between traditional bulk materials and 2D materials; i) the near-atomic thickness of 2D materials is covalently bonded with a dangling bond free surface, and ii) mono- or few-layer of 2D materials can be exfoliated from bulk 2D crystals due to their weak van der Waals (vdW) force interaction [48–50]. Since the single graphene layer has been successfully exfoliated from bulk graphite, the other 2D materials, including transition metal dichalcogenides (TMCs), hexagonal boron nitride (h-BN), and black phosphorus (BP), also have been received tremendous academic and research focus. Among these 2D materials, the 2D TMCs and BP have semiconducting properties. They have been explored to develop a new generation of switching devices based on their outstanding electrical properties [51–53]. Especially, the physical properties of 2D TMCs can be determined by the constituent transition metal and chalcogen atoms, for example, metallic (NbS_2_, TiS_2_), semi-metallic (PdTe_2_, PtTe_2_), and semiconducting (MoS_2_, WS_2_) [54–57].

Furthermore, there are many polymorphs in 2D TMCs, including MoS_2_ polymorphs such as 2H-, 1T-, and 1T′-MoS_2_ that have different atomic structures and physical properties and can be used as an active layer in memristive and neuromorphic devices. To maintain the device’s performance in memristive applications, precise control of phase change and its reliability is critical. Using lithium ions (Li^+^) and an external electric field, Zhu et al. demonstrated a reversible phase transition from 2H-phase (semiconducting) to 1T'-phase (metallic) MoS_2_ [58]. MoTe_2_ is another well-known example of phase transition in 2D TMCs. Zhang et al. investigated phase-change resistive memory devices by studying the phase transition of MoTe_2_. When an electric field is applied, the semiconducting 2H-phase MoTe_2_ transitions to a distorted 2H-phase with electronic properties intermediate between the semiconducting 2H- and metallic 1T′-phases [59]. These phase transition properties open up new possibilities for developing phase-change-based memristor devices. In the case of more complicated polymorphs in indium selenides, such as the α-, β-,γ-, κ-phases of In_2_Se_3_ and even InSe with physical diversities such as ferroelectrics and piezoelectrics [60, 61], to control the ferroelectric domains, the ferroelectric memristor utilizes polarization switching dynamics. The electrical pulses over their coercive voltage can be gradually switched to the ferroelectric domains, resulting in a change in the device resistance state from a high-resistance state (HRS) to a low-resistance state (LRS). One of the research topics to expand the memristive and neuromorphic research area is fast and reliable domain switching in 2D ferroelectrics. Due to their diverse functionalities and quantum confinement effect, the near-atomic thickness nature of 2D TMCs also provides a new type of solution to overcome the bottleneck of Moore’s law.

**Fig. 2:**
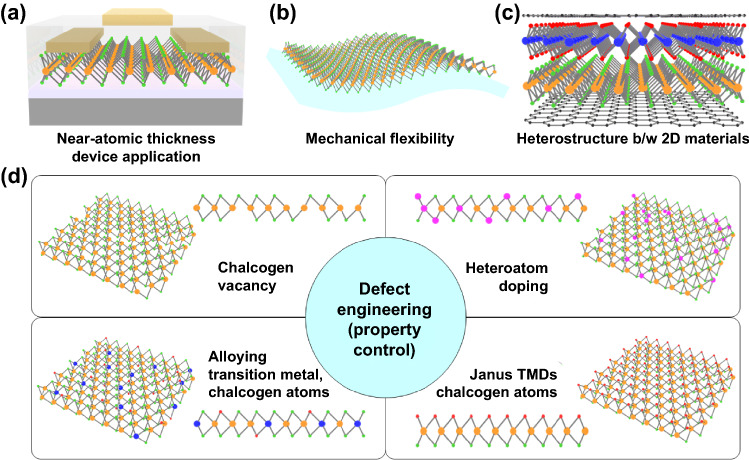
Promising aspects of 2D TMC materials for memristor-based neuromorphic devices; **a** device fabrication using near-atomic thickness, **b** mechanical flexibility, **c** reduced contact resistance owing to heterostructure between 2D materials, **d** property controlling by various defects engineering including chalcogen vacancies, heteroatom doping, alloying, and Janus structures

The 2D TMCs are considered as one of the possible candidates to realize the ideal neuromorphic device which has the following requirements; (1) fast switching process, (2) high-density integration, and (3) lower energy consumption [62–64]. Owing to their extremely low thickness, it is advantageous to make a highly integrated device architecture and achieve fast switching, resulting in lower power consumption (Fig. 2a). There are several operation mechanisms of 2D TMCs in neuromorphic devices, including memristive switching, phase change, ferroelectric behavior, and their unique heterostructures, etc. In the vertical memristive devices, the 2D materials can be reduced to a sub-nanometer scale to achieve high-density device integration and low operation voltage. From these types of devices, the multiple resistance states can be modulated by forming and disappearing conductive filaments through defects and ion migrations by external bias [65–67]. Through these memristive switching mechanisms and their structural advantages, the 2D TMCs have been considered as an excellent channel material to realize low power consumption (highly integrated) and reliable operation [68]. Furthermore, the excellent mechanical features of 2D materials enable applications into flexible devices (Fig. 2b) [64, 69, 70]. From these aspects, the 2D TMCs-based wearable or highly flexible optical/electrical synaptic device applications are promising. The most attractive feature of 2D TMCs is the creation of the vdW heterostructures (Fig. 2c). The integration of 2D materials enables overcoming the limitation of a single layer of TMCs. Furthermore, the vdW stacking features have a great advantage for making a contact between 2D materials without leakage current or failure [71, 72]. For example, it is well known that semi-metallic TMCs or graphene layers can be used as an electrode, and semiconducting TMCs can be used as a channel in various types of device applications to reduce device failure and leakage current [73, 74]. Especially, the vdW heterostructures can be utilized to achieve desired synaptic characteristics and develop various types of electronic/optoelectronic synaptic characteristics via gate tunability and divers energy band configurations [75, 76].

The cation-based resistive switching device can be realized through defect engineering (Fig. 2d) by redistributing atomic vacancies. H_2_ etching, surface plasma treatment, thermal annealing, and other methods are used to generate transition metal or chalcogen vacancies [77, 78]. When compared to pristine 2D TMCs, the surface atomic vacancies facilitate the various charge transfer mechanisms. The Miao group demonstrated graphene/MoS_2−*x*_O_*x*_/graphene memristors that can operate reliably with resistive switching over a wide temperature range. The electroforming process changed the concentration of sulfur vacancy in partially oxidized MoS_2−*x*_O_*x*_ layers, resulting in different resistance states such as the LRS and HRS under the applied bias [79]. Furthermore, an external electric field can generate a vacancy-induced conducting path with conductive-filament-like behavior, similar to resistive switching devices. Phase engineering can be used to tune the electrical properties of 2D TMCs. MoS_2_ can, for example, be transformed from trigonal (2H) to octahedron (1T) via Li^+^ ion intercalation, charge transfer, metallic atom insertion, and stress inductions [80, 81]. These phase transitions are also regarded as extended opportunities in various 2D TMCs. The phase transition and physical properties of 2D TMCs can be modulated by heteroatom doping. For example, after Re substitutional atom doping into MoS_2_, the 2H-phase MoS_2_ can be changed to 1T-phase MoS_2_ [79]. Another study, doping of Re atoms, successfully demonstrated the doping of Re atoms into MoS_2_ during the CVD growth process. The electrical property of the Re-doped MoS_2_-based field-effect transistor has been verified to be degenerated *n*-type by evidence of a large shift in threshold voltage [82]. Unlike Re atoms, tungsten (*W*) is the ideal element for creating isoelectronically doped TMC semiconductors. Li et al. used CVD to create W-doped MoSe_2_ layers. One of the major advantages of such heteroatom-doped or alloyed TMCs is that the amount of doping concentration can modulate deep trap states within the bandgap [83, 84]. Furthermore, structural distortion and photon scattering can be used to control the optical properties, allowing the recombination dynamics to be controlled. Because of these advantages, heteroatom doping and metal or chalcogen atom alloying have a high potential for extending the use of 2D TMCs as active materials in memristive and neuromorphic devices. In terms of determining electronic and optical properties in 2D materials, structural symmetry-breaking is critical. The breaking of out-of-plane mirror symmetry, Janus structuring, the creation of new types of defect-engineered 2D TMC layers, and the demonstration of piezo- or ferroelectric new functionalities are all possible as a result of the breaking of out-of-plane mirror symmetry. Lu et al. demonstrated the MoSSe Janus monolayer and characterized the symmetry breaking in their atomic structure using a simple plasma and selenization process [85]. They went on to discover that the synthesized MoSSe layer has high piezoelectricity. There are currently only a few studies for Janus 2D TMCs, so more research is needed to investigate their physical and chemical properties, as well as to find a way to extend to memristive and neuromorphic applications.

Since some 2D TMCs have been reported their photo-responsivity or ferroelectricity, the light and electrical polarization can be used for external stimuli rather than electrical voltage pulses [86–88]. For the photo-responsivity, the consecutive light pulses with the specific wavelength can be used for stimulus. The functional integration between optical and electrical stimuli can be a possible candidate to enable power-efficient pattern recognition. Through an optic neural network formed by these optic-neural synaptic devices, the colored and color-mixed pattern recognition functionality can be emulated [89]. Another new feature is ferroelectric analog synaptic devices which can achieve high-speed, highly integrated devices, and low power consumption [90, 91]. The 2D TMC-based ferroelectric have been reported in recent years and it has strong spontaneous polarization to be modulated by an external electric field. The ferroelectric domains can be delicately controlled and the channel conductance is also gradually controlled by the polarization of ferroelectric materials [92–94]. These characteristics can be the breakthrough to resolve issues such as low on/off ratio, nonlinear weight changes, and variations of electrical properties. The new functionality related to ferroelectricity will be discussed in the following section for more details. For these reasons, the 2D TMCs are a highly potential material group for the development of neuromorphic applications in terms of scalability, learning, and energy efficiency.

#### Two-D Ferroelectrics: Additional Functionality

Because of its polarization properties and potential applications, non-centrosymmetric materials are an important research area. Researchers are interested in 2D layered materials with non-centrosymmetric structures for nanoscale electromechanical and electronic devices. Ferroelectricity is a property of spontaneous electric polarization that can be controlled by an applied external electric field, making it ideal for nonvolatile random access memory (RAM), actuators, and stress sensors, among other applications. Perovskite oxides have been proposed as a possible candidate for realizing ferroelectric RAM (FeRAM) with higher storage density and lower power consumption based on their nonvolatile switching properties. Due to the cooperative behavior of electric dipoles and depolarization effects from uncompensated interfacial charges, there is a critical thickness of around tens of nanometers in traditional 3D perovskite oxides to maintain stable ferroelectricity. Apart from thickness issues, oxygen vacancies are unavoidable during high-temperature synthesis and nanostructure fabrication, causing the measurement of intrinsic ferroelectric properties to be disrupted [95, 96].

2D ferroelectrics have recently been investigated for their unique physical properties and the origin of their ferroelectric behavior. The ferroelectricity of 2D materials has been predicted for a long time, but due to the fundamental constraint of size effects and the dead-layer effect originating from interfacial strain and local chemical environments [97, 98], it is difficult to demonstrate their realistic ferroelectricity. In recent years, the thickness of 2D layered or vdW materials has been reduced to near-atomic levels, resulting in strong in-plane bonds rather than layer coupling [96]. In recent reports, several 2D ferroelectric materials have been theoretically predicted and experimentally verified. 2D ferroelectrics can be classified as in-plane ferroelectric semiconductors (group IV monochalcogenides; SnS, SnSe, GeS, and GeSe), out-of-plane ferroelectric semiconductors (*d*1T-MoTe_2_, WTe_2_, and CuInP_2_S_6_), and intercorrelated ferroelectrics (III_2_-VI_3_ compounds, e.g., In_2_Se_3_) [94, 99, 100]. The ability of layered materials to retain ferroelectric properties to the nanometer range, in contrast to 3D perovskite oxides, is due to their weak interlayer coupling, which stabilizes individual layers from out-of-plane perturbations. The off-centering of a sublattice of composing atoms and the rotation of sublattices at their atomic structures are the sources of local polarization in 2D ferroelectrics. The movement of Cu and In sublattices in CIPS thin films under the curie temperature (*T*_c_), for example, causes spontaneous polarization [101]. Furthermore, it has the lowest energy with a symmetry-breaking Zincblende/wurtzite crystal structure in the case of -In_2_Se_3_. The intercorrelated (out-of-plane and in-plane) ferroelectric switching behavior in -In_2_Se_3_ is due to the lateral movement of Se atoms in the middle layer [102].

Despite numerous attempts to understand the origins of ferroelectricity in some 2D materials, determining the exact mechanism that causes spontaneous polarization remains difficult. 2D ferroelectrics are one of the new features that will help neuromorphic devices overcome their current bottlenecks in terms of device integration density and low power operation. 2D ferroelectrics have been proposed as one of the possible key materials for a new-generation nonvolatile memory device due to their strong remanent polarization and lack of depolarization effect [103–105]. Furthermore, narrow bandgap 2D ferroelectrics are advantageous for optoelectronics but are uncommon in traditional ferroelectric ceramic oxides. 2D ferroelectrics, such as α-In_2_Se_3_ and SnS, have a tunable bandgap that can be used in a variety of optoelectronic devices, including photodiodes and energy conversion devices. Most 2D ferroelectrics are semiconductors with an appropriate bandgap, according to theoretical and experimental reports, so devices similar to ferroelectric field-effect transistors (FeFETs) can be made without an additional semiconductor channel layer, resulting in improved device integration.

Because of the advantages of 2D ferroelectrics, FeFET devices can be used to create ferroelectric analog neuromorphic devices with a variety of device structures. Despite these advantages in 2D ferroelectrics, due to the difficulty of large-area production and the low spontaneous polarization value, there is still a long way to go to achieve high-performance 2D ferroelectric-based electronic/optoelectronic devices.

### Operation Mechanisms

**Fig. 3:**
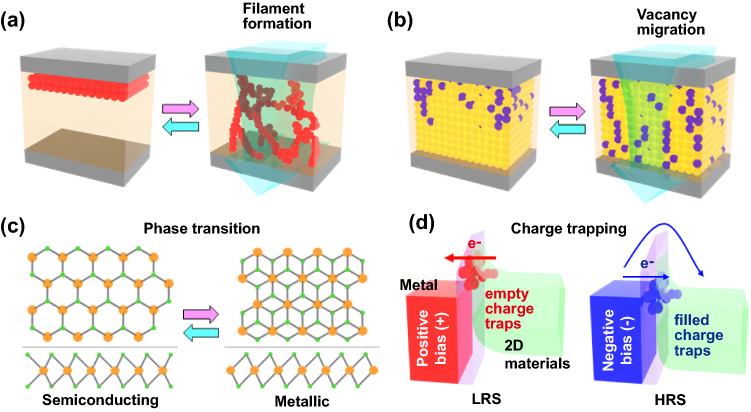
Representative working principles of memristor devices based on 2D TMC materials and that of schematic illustrations; **a** filament formation, **b** vacancy migration, **c** phase transition, and **d** charge trapping

Numerous 2D TMCs materials with various polymorphisms possess various inherent advantages in structural, electrical, and phase controllable characteristics [56, 57, 106]. In particular, precisely controllable phase change and atomic-scale ultrathin structure enable memristive and neuromorphic devices to have excellent reliability/ reversibility, fast switching, low power consumption, and high-density device integration [107, 108]. Therefore, 2D TMCs have received a lot of attention as prospective advanced material platforms for future memristive and neuromorphic applications. Plenty of material systems for memristive and neuromorphic devices can be built based on the various configuration of over 80 types of TMCs represented for M_x_C_y_ (M; transition metals, e.g., Mo, W, Sn, Hf, Zr, and C; chalcogen atoms, e.g., S, Se, Te) and diverse crystal systems. Despite the structural similarity, the specific carrier transport mechanism, which is determined the fundamental device working principle of memristive and neuromorphic behaviors, may depend on the atomic components. In the following section, we highlighted (summarized) the four well-known principles represented for memristive and neuromorphic devices; (1) conductive filament formation, (2) vacancy migration, (3) charge trapping/de-trapping, (4) phase transition, as illustrated in Fig. 3. Furthermore, the performances of memristors and neuromorphic devices are summarized in Table 1 including device structure, fabrication method, operating voltage, and energy consumption.

**Table 1:** Memristor and neuromorphic devices based on 2D TMCs

Device	Working principle	Structure	Growth/fabrication method	Switching/SET voltage (V)	Pulse width (ms)	Energy per spike (J)	Refs.
Memristor	Conductive filament formation	Ag/MoS_2_/Ag	CVD growth	0.35–0.4	1	–	[109]
Memristor		Cu/MoS_2_/Au	MOCVD growth	0.1–0.2	1	–	[107]
Memristor		Ni/Au/MoS_2_/Gr	CVD growth	3	5	–	[110]
Memristor		Ag/ZrO_2_/WS_2_/Pt	CVD growth	0.4	100 ns	–	[111]
Memristor	Vacancy migration	Pd/WS_2_/Pt	Liquid exfoliation	2	500 ns	~ 26 fJ	[112]
Memristor		Gr/ MoS_2−*x*_O_*x*_ /Gr	Mechanical exfoliation	3.5	1 μs	–	[63]
Memristor		Ag/ WO_3−*x*_ /WSe_2_/Gr	Mechanical exfoliation	3	10	~ 20 pJ	[113]
Memristor		Ag/MoO_x_/MoS_2_/Ag	Langmuir–Blodgett coating	0.15	50 μs	–	[114]
Memristor	Phase transition	Au/MoS2/Au	Mechanical exfoliation (lithiated MoS_2,_ 2H → 1 T)	4	1	–	[58]
Memristor		Ti/Ni/MoTe_2_/Ti/Au	Mechanical exfoliation (2H → 2H_d_)	0.066	–	–	[59]
Memristor		Ag/MoS_2_/Ag	Mechanical exfoliation (2H → 1 T)	2.3	10 ns	–	[115]
Synaptic transistor	Charge trapping/de-trapping	Cr/Au/h-BN/Gr/h-BN/MoS2/Al_2_O_3_	Mechanical exfoliation	6	250	64 pJ	[116]
Optic-neural synaptic		Ti/Au/WSe2/WCL/h-BN/Pt/Au	Mechanical exfoliation	0.3	10	66 fJ	[89]
Synaptic transistor		Cr/Au/MoS_2_/h-BN/Gr	Mechanical exfoliation	3	0.1 s	5 fJ	[117]

#### Conductive Filament Formation

Conductive filament formation is a major working mechanism in conventional resistive switching memory devices, which generally adopt a two-terminal structure of memristive materials sandwiched between the top and the bottom electrodes [118–120]. Both memristive and artificial synaptic characteristics can be emulated via the formation and control of the filaments by adjusting the input voltage pulse or bias sweep as shown in Fig. 3a. Dev et al*.* reported a vertically aligned two-terminal Ag/MoS_2_/Au device for threshold switching memristor (TSM)-based artificial neurons [109]. The resistive switching is attributed to the formation of conductive filaments by silver ions from the Ag electrode in the electrochemically activatable MoS_2_ layer under an electric field. Without the electric fields, the filaments spontaneously collapse into a spherical shape to minimize the interfacial energy by the self-rupturing effect of Ag ions. When the applied bias becomes higher, the filament becomes more robust, allowing the device to remain in the low resistance state (LRS), a “fully formed state.” After that, the filament has disappeared when the reverse bias is applied to the device, leading to the device undergoes to a high resistance state (HRS). These volatile behaviors satisfy the basic requirements of artificial neurons [121, 122].

**Vacancy migration** In addition to the metal-ion or oxygen-vacancy filament formation, another resistive switching mechanism is based on vacancy migration, as displayed in Fig. 3b. There are some intrinsic vacancies in 2D TMCs materials and more could be generated by external stimuli such as electrical fields [123–125]. Therefore, according to vacancy distributions, the resistance states of the device can change from LRS to HRS depending on the applied bias [67, 114]. Miao et al*.* demonstrated a robust memristor of vertically integrated graphene/ MoS_2−*x*_O_*x*_ /graphene with high thermal stability [63]. The MoS_2−*x*_O_*x*_ layers as resistive switching materials were obtained by oxidation of MoS_2_ through ambient air annealing at 160 ˚C. In the pristine MoS_2−*x*_O_*x*_ layer, most of the sulfur vacancies are occupied by oxygen with a uniform atomic distribution. As electroforming progresses during the on process, more sulfur vacancies are generated in the pristine MoS_2−*x*_O_*x*_ layer and form conductive channel regions with high concentrations of sulfur vacancies, resulting in significant changes in composition. And following the “off” process, existing sulfur vacancies in the channel area are occupied by oxygen ions from the surrounding region. The authors also showed that the loss of sulfur elements can be caused by the thermo-migration effect due to Joule heating.

#### Phase Transition

2D TMCs comprised of dozens of ultrathin layers generally possess several different stable phases determining their properties [126–128]. A controllable phase change characteristic in TMCs has been extensively investigated for the feasibility of a memristive and neuromorphic application (Fig. 3c) [59, 129]. Cheng et al. reported the odd-symmetric vertical memristor, which has a simple Au/MoS_2_/Au structure [115]. Though the device has a similar structure with the conventional vertically configurated metal/insulator/metal (MIM) structures employed for bulk materials, the resistive switching behavior is governed by the phase transition of MoS_2_ nanosheets. It is demonstrated that the 2H-phase bulk MoS_2_ showed ohmic properties, whereas the 1T-phase disjoined MoS_2_ exhibited a specific memristive response resulting in conductance changes along with the voltage variation. The electrical conductivity of MoS_2_ is about 10^7^ times higher in the 1T-phase than in the 2H-phase [97, 130]. The applied electric field induces lattice distortion, facilitating the delocalization of 1T phase electrons, significantly increasing the electrical conductivity, resulting in severe resistance changes. In addition, the resistance gradually changes as the voltage sweep is applied in sequence, satisfying the primary synaptic properties.

#### Charge Trapping/Detrapping

Owing to a stable and reversible physical operation, the charge trapping/de-trapping mechanism has been widely used to build various memristive and neuromorphic devices [131–133]. In various 2D van der Waals materials, resistive switching mechanism ascribed to charge trapping and de-trapping has been widely reported [89, 134, 135]. Charge trapping and de-trapping of electrons arise from the interface between the electrode and 2D TMC layers, the so-called weight control layer (Fig. 3d). Vu et al*.* developed two-terminal floating gate memory composed of vertically aligned monolayer MoS_2_/hexagonal boron nitride (h-BN)/monolayer graphene [136]. For source and drain, two Cr/Au electrodes were located on the monolayer MoS_2_ channel. In this device, memristive characteristics are adjusted by tunneling-induced charge trapping/de-trapping at the graphene floating gate, which acts as a weigh control layer through the tunneling barrier of h-BN. The charge trapping and de-trapping process in the graphene layer have been carried out via drain voltage control. Electron tunneling occurs from drain to graphene in a sufficiently large potential drop regime between them, whereas the electron tunneling into MoS_2_ or the source cannot arise owing to the small potential drop at the junction. The negative electrical field generated by trapped electrons depletes the most of electrons in the MoS_2_ channel, leading to HRS vice versa; in the case of LRS, it is holes, not electrons.

## Recent Advances in 2D TMC-based Memristors for Neuromorphic Computing

2D materials have emerged as resistive switching materials with great potential for fabricating ultrathin memristive synapses with high-density device integration and reliable operation. Due to the near-atomic thickness of 2D materials, device miniaturization, high-density crossbar arrays, and heterostructured devices via van der Waals layer coupling are all possible. Neuromorphic devices based on electrical stimuli have recently been reported to mimic the various functionalities of biological synapses [137, 138, 139]. Based on the previously described operation mechanisms, we will introduce memristive and neuromorphic devices based on 2D TMC in this section. Resistive switching properties, as well as several types of synaptic plasticity that mimic biological synapses, will be also discussed.

### Memristor-based Neuromorphic Computing Applications

**Fig. 4:**
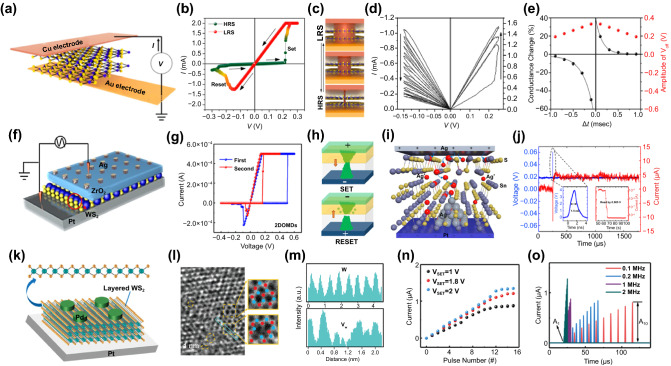
TMDs-based artificial synapse. **a** Vertical Cu/MoS_2_/Au memristors based on conductive filaments formation. **b** Typical I–V curve of resistive switching. **c** Schematics of the cumulative area change of Cu filaments causing LRS to HRS transition. **d** Bipolar and analog switching characteristic as an artificial synapse. **e** Demonstration of STDP behavior. Reproduced with permission from Ref. [107]. Copyright 2019, Nano Letters. **f** Structure of Ag/ZrO2/WS2/Pt memristor device. **g** I − V characteristics of Ag/ZrO2/WS2/Pt heterostructure. **h** Schematic of the SET and RESET process. Reproduced with permission from Ref. [111]. Copyright 2019, American Chemical Society. **i** Illustration of the ion dynamics in Ag/SnS/Pt memristor. **j** Ultrafast switching curve of the device with 4 V/1.5 ns voltage pulse. Reproduced with permission from Ref. [140]. Copyright 2021, Nano Letters. **k** Schematic of Pd/WS_2_/Pt memristor operated by atomic-vacancy formation and migration. **l** TEM images of WS_2_ films in the LRS. **m** Line profile for W atoms; the upper (HRS) and lower images (LRS). **n** Pulse modulation by changing input voltage amplitude. **o** Response currents for pulse trains of 4 different frequencies (0.1, 0.2, 0.5, and 1 MHz). Reproduced with permission from Ref. [112]. Copyright 2019, Small (Color figure online)

The adoption of 2D materials in a vertical memristor device enables scaling down to a thickness of 10 nm or less, achieving low switching voltage and high integration density, thereby can be useful for low-power applications [141, 142]. A vertically aligned Cu/MoS_2_ bilayer/Au structured memristor fabricated by Xu et al*.* presents resistive switching characteristic attributed to Cu filament formation through MoS_2_ double layer (Fig. 4a) [107]. The electrochemical metallization of Cu ions in atomic-scale thickness enables the bipolar switching operated under an exceedingly low set voltage of 0.2 V combined with a low activation barrier of Cu ions diffuse (Fig. 4b) [143]. The devices also exhibit analog resistance switching characteristics, allowing artificial synapse applications. The cumulative Cu filament cross-sectional area varies with consecutive DC bias sweep, resulting in a gradual change in conductance state by three times (Fig. 4c, d). The multi-step potentiation and depression of synaptic weight are modulated by positive and negative sequential programming voltage pulse trains, respectively. In addition, the very first demonstration of STDP in 2D TMCs-based memristors was carried out. In neuroscience, the advanced learning rules for competitive Hebbian learning are generally described as spike-timing-dependent plasticity, which represents a change in synaptic weights as relatively dynamic and temporal reciprocal activities between pre- and postsynaptic spikes. Indeed, for spatiotemporal processes in the biological synapse, when a spike signal reaches both anterior and posterior neurons simultaneously, the resulting synaptic weight between neurons depends on the relative time delay between spikes. Therefore, as | Δ*t* | is smaller, the superposition of the pre- and the postsynaptic pulse reinforces, and the influence of the effective voltage pulse increases, and thus, the degree of conductance change becomes larger (Fig. 4e). Demonstrating STDP with low voltage spikes gives the potential for this work to be considered as a feasible candidate for artificial synaptic devices in neuromorphic systems.

In conductive filament-mediated memristive devices, the random distribution of conductive filament formation is a significant issue affecting device uniformity and stability [114]. Yan et al. fabricated a memristive device using a ZrO_2_/WS_2_ heterojunction active layer to control the formation and rupture process of conductive filaments (Fig. 4f) [111]. The switching properties of the heterojunction structure Ag/ZrO_2_/WS_2_/Pt device outperform those of the single-layer Ag/WS_2_/Pt and Ag/ ZrO_2_/Pt devices (Fig. 4g). This can be explained by the limited rupture and regeneration of Ag conductive filaments in the ZrO_2_/WS_2_ bilayer interfacial region (Fig. 4h). Such localized filament formation in hetero-bilayers can considerably improve the uniformity and stability of resistive switching capabilities by reducing the unpredictability of the nucleation and rupture process of conductive filaments. In filament-mediated memristive devices, it is believed that the occupation of vacancies by oxidized metal ions is the origin of resistive switching [144]. Here, the diffusion of metal ions forming conductive filaments can be assisted by vacancy migration. Lu et al*.* reported Ag/SnS/Pt memristor devices that allow for robust facile Ag filament formation attributed to the presence of Sn vacancies (Fig. 4i) [140]. In contrast to most TMCs, which are intrinsically n-type due to chalcogen vacancies, SnS is an intrinsically p-type semiconductor due to Sn vacancies [145]. Theoretically, Sn vacancies have more favorable energy for metal ion migration than chalcogen vacancies. When compared to n-type transition metal chalcogenides, the presence of cation vacancies and van der Waals gaps lowers the activation barrier for Ag ion migration, allowing for low energy and ultrafast switching of p-type SnS. Attributed to the above, the Ag/SnS/Pt device exhibits exceptional metrics such as a switching voltage of 0.2 V, fast switching of less than 1.5 ns, and low power consumption of less than 100 fJ per switch. (Fig. 4j). Without filament formation, a memristor device with the resistive switching mechanism based on vacancy formation and the electron hopping across the vacancies between tungsten and sulfur in 2H-phase WS_2_ was also reported by Yan et al*.* (Fig. 4k) [112]. In this vertically aligned Pd/WS_2_/Pt device, the resistive switching characteristics are explained by a trap-assisted tunneling mechanism. The TEM investigations revealed more S and W vacancies in the WS_2_ nanosheet in the LRS than the pristine one (Fig. 4l, m). When the voltage is applied, the internal temperature increases because of both the Joule-heating and the thermophoresis effect, leading to additional vacancy generation in S and W sites. As a result, the defect concentration increased at LRS, the reduced distance between the vacancies facilitates electron hopping among them, and hence, the total conductance increased. It suggests that both W and S vacancies in WS_2_ can serve as dopants and migrate under the bias applied. Various synaptic plasticities, such as spiking voltage-, spiking frequency- and spiking width-dependent plasticities, were emulated with different programming pulse schemes (Fig. 4n, o) When a series of pulse trains are input to the device, the current gradually increases, eventually reaching an upper limit. This upper limit increases with larger pulse amplitudes or smaller pulse intervals. The spiking voltage-dependent plasticity was clearly shown with the larger saturation current with a 2 V pulse than a 1 V pulse. Further, the spiking frequency-dependent plasticity, which tends to increase the maximum current value as the applied pulse frequency increases, was also demonstrated. Interestingly, in these modulations, the device current remains less than 2 μA, showing low power consumption, which can be suitable for energy-efficient neuromorphic applications.

**Fig. 5:**
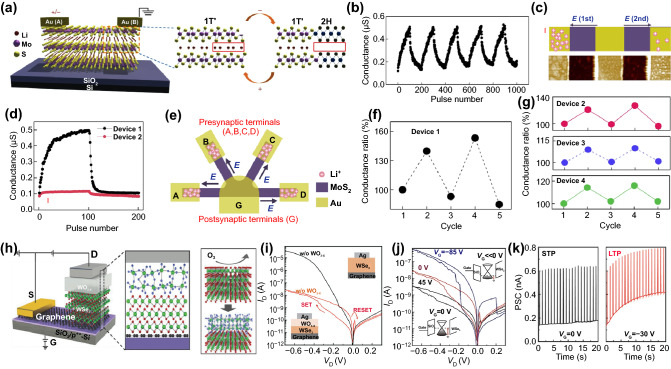
Interactive and regulatory synaptic devices. **a** Electric field controllable MoS_2_ devices depending on local 2H–1 T' phase transitions. **b** Synaptic potentiation and depression by programming pulse. **c** Schematics of Li^+^ ions distribution (top), and AFM height images (bottom) and **d** synaptic potentiation and depression showing synaptic competition. **e** Schematic of the four electrodes network at the initial condition for synaptic cooperation. **f** Potentiation and depression in device 1 and **g** conductance changes of non-stimulated devices 2, 3, and 4 in response to device 1. Reproduced with permission from Ref. [191]. Copyright 2019, Nature Materials. **h** Illustration of heterojunction structures composed of WO_3–x_ memristor and WSe2/graphene synaptic barristor. **i** I_D_–V_D_ characteristics with (red line) and without a WO_3–x_ layer (black line). **j** The gate-tunable I_D_–V_D_ switching curves depending on the Schottky barrier height. **k** STP to LTP transformation induced by V_G_ change. Reproduced with permission from Ref. [113]. Copyright 2018, Advanced Materials (Color figure online)

For reliable memristor applications, not only precise control over the phase change duration but also the reversibility of the process is important. Layered 2D materials such as MoS_2_ present highly anisotropic ionic transport properties upon phase change, facilitating efficient ionic modulation and ionic coupling among devices based on coupled ionic-electronic effects. Lu group demonstrated a memristor-based on electric field-controlled reversible phase transition of MoS_2_ accompanied by Li-ion migration [58]. Natural MoS_2_ materials primarily exist in the 2H-phase [57]. When the MoS_2_ film is lithiated by intercalation of lithium ions between the sheets, a 2H- to 1T-phase transition occurs, and the conductivity increases by more than three orders of magnitude compared to the pristine MoS_2_. The device using this phase transition exhibits reliable memristive properties in which the local low/high lithium-ion concentration driven by an electrical field induces phase transition to the semiconducting 2H-phase/metallic 1T′-phase, respectively (Fig. 5a). Direct evidence of Li^+^ ion intercalation was corroborated by the cross-sectional high-resolution transmission electron microscopy (HRTEM) at HRS and LRS. The MoS_2_ sheets under the Au electrode expand in the LRS due to the Li-ion penetration, whereas it contracts in the HRS. A probable explanation for the 2H- to 1T′-phase transition is that the intercalation of Li^+^ ions destabilizes the 2H-phase while donating electrons to the 4d orbital of Mo elements. This theory was confirmed by X-ray photoelectron spectroscopy (XPS), which showed that the lithiated MoS_2_ film contains a fraction of the Mo^3+^ converted from Mo^4+^. More interestingly, this work implemented the emulation of artificial synapses as well. The reversible migration of Li^+^ ions resulting from consecutive bias applied allowed it to realize the synaptic plasticity of weight potentiation and depression (Fig. 5b). Remarkably, the devices can be directly connected via local ion exchange which can be seen as synaptic competition/cooperation in biology referring to the interaction that leads adjacent synapses to different/similar learning outcomes upon signal input from multiple terminals [146]. The synaptic competition can be demonstrated by the limitation of available Li^+^ ions in the Li_x_MoS_2_ film. Even when the identical pulses were alternately applied to two adjacent synapses, significant potentiation of synaptic weight was observed only in one synapse due to the biased Li^+^ ion migration in a limited amount (Fig. 5c, d). Besides, synaptic cooperation is emulated by showing that the potentiation of one synaptic device facilitates the potentiation of a neighboring synaptic device. At a network composed of four synaptic devices based on Li_x_MoS_2_ that share one electrode in common (Fig. 5e), it is shown that programming a target device in the network cooperatively affects the synaptic plasticity of adjacent non-target devices via ionic coupling effects (Fig. 5f, g). This phenomenon can be utilized to emulate plasticity modification using neighboring synapses, a reinforcement strategy following the Hebbian learning rule. Moreover, it can be useful for effectively implementing synaptic interactions in artificial neural networks at low energy consumption.

Unlike the structures previously discussed, the heterojunction-architecture presented in this part not only works on different mechanisms but also offers additional further functions such as flexibility and heterosynaptic operation. Huh et al. reported vertical three-terminal synaptic architecture, named as a “synaptic barristor,” consisting of a memristive WO_x_ and a WSe_2_/graphene Schottky diode as barrier tunable barrister (Fig. 5h) [113]. Typical bipolar resistive switching behaviors were observed at low drain voltages of less than 0.4 V, which originates from the ultrathin amorphous WO_3−*x*_ layer grown by monolithic oxidation of the WSe_2_ layer (Fig. 5i). The switching would be attributed to the migration of induced oxygen vacancies induced in the vicinity of the interface of the top Ag electrode with WO_3−*x*_, which is formed by partial oxidation of Ag. More interestingly, due to the electrostatically gate-tunable Schottky barrier at the junction of WSe_2_ and graphene, the resistive switching phenomena are substantially adjusted by varying the gate voltage (Fig. 5j). Moreover, such dynamic barrier tunability enables the conversion between STP and LTP, intrinsic synaptic plasticity, by only adjusting the gate voltage while source–drain input spikes are maintained (Fig. 5k). The electrostatic tunability of the synaptic weight is similar to the heterosynaptic plasticity of biological synapses, which is an energy-efficient strategy for weight strengthening induced by third modulatory neurons [147, 148]. The synaptic barrister based on heterojunction architecture with electrostatic tunability potentially provides considerable power-saving benefits for weight modulation generated by third regulatory neurons.

**Fig. 6:**
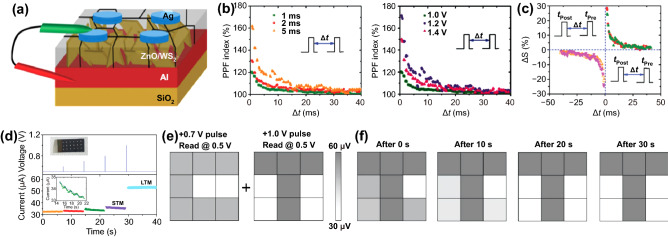
Memristor devices mediated by charge trapping and de-trapping. **a** Schematic structure of the Ag/ZnO/WS_2_/Al memristor. **b** PPF decays as a function of pulse interval (Δ*t*) at different pulse amplitude and different pulse widths. **c** Experimental results for STDP. **d** The STM to LTM transition and the conductance variation by increasing input pulse voltage from + 0.7 to + 1.0 V. **e** Images of the letters “C” and “T” memorized in STM and LTM modes, respectively. **f** The measured current history of the 3 × 3 arrays from right after to 30 s after the written processes. Reproduced with permission from Ref. [149]. Copyright 2019, Advanced Electronic Materials (Color figure online)

Similar to the previous study which used an electrostatically gate-tunable Schottky barrier, Chen et al*.* demonstrated a van der Waals heterostructure device (Fig. 6a) with flexible time-tailoring capability, which can facilitate integrated STP to LTP modulation [116]. The time-tailoring capability of a heterostructure is determined by the volatile and nonvolatile functional layers that are designed by tailoring memory time. The volatile characteristic is dominated by top gate operation, while the nonvolatile characteristic is dominated by back gate operation [150]. Here, the memory operation mode and the thickness of the tunneling layer are the major determinants of memory properties. When the device is in top gate operation, electrons from the top electrode are injected into the graphene layer at a negative voltage through a 7 nm tunneling layer, hBN (Fig. 6b). However, due to the thinness of the tunneling layer, tunneling carriers cannot be stored in graphene for an extended period. The heterostructure displays nonvolatile characteristics at back gate operation mode by utilizing the capabilities of the high-k Al_2_O_3_ layer of 23 nm thick. At high positive voltages, electrons in the MoS_2_ sheet tunnel hBN and become trapped in the Al_2_O_3_ layer (Fig. 6c). Consequently, the accumulation of trapped electrons in the Al_2_O_3_ layer can partially shield the electric field at the back gate, resulting in a positive shift of the threshold voltage. By stacking both volatile and nonvolatile functional layers, basic types of memory including short-term and long-term memory are integrated into the device and the transition between these memory types is adaptable. When a small bias is applied to the back gate, the electrons are temporarily trapped by a shallow trap in Al_2_O_3_ layer near the interface. As the bias increases, the electrons tunnel into deeper traps and are stored for a longer period of time. Therefore, memory type transition from STP to LTP is made possible by applying a continuous pulse (Fig. 6d).

In 2D vdW synaptic devices, the length of the tunneling barrier can be increased or decreased on the trapped or de-trapped electrons. Kumar et al. reported memristive and neuromorphic devices composed of vertically grown WS_2_ layer and ZnO (Fig. 6a) [149]. The interlayer separation between WS_2_ and ZnO layers serves as an effective porous medium allowing the ZnO to grow with defects. The interfacial region of ZnO, the very contiguous to WS_2_ layer, consists of highly non-stoichiometric oxygen-deficient condition, as confirmed by depth-dependent XPS measurements. In this defective interfacial layer, the randomly distributed oxygen vacancies play the role of charge trapping/de-trapping centers. Hence, as the programming pulse is applied, injected charge carriers fill the defects, causing internal conductance change. To realize the relevant neurological functions in synapses, various plasticities were demonstrated under diverse electric stimuli. Paired pulse facilitation (PPF) in neuroscience is a form of short-term plasticity manifested as strengthening the second excitatory postsynaptic potentials (EPSPs) in rapidly evoked two close spikes [151, 152]. It is shown that the PPF index decreases exponentially toward 100% as the inter-spike time Δ*t* increases from 0.1 to 40 ms, in agreement with the biological synapse (Fig. 6b). Furthermore, it can be seen that the change in the second EPSP at small Δ*t* tends to enhance with increasing spike width and voltage. Additionally, they also demonstrated the LTP, LTD, and STDP to show emulation of the synaptic device using WS_2_/ZnO heterostructure (Fig. 6c). Unlike conventional digital memory, human memory is commonly represented as short-term memory (STM) and long-term memory (LTM) [153, 154]. The STMs are lost within minutes, whereas LTMs are permanent changes, lasting from hours to years or longer [155]. In this synaptic device, a transition from STM to LTM is observed by increasing the pulse voltage from + 0.7 to + 1.0 V (Fig. 6d). Image programming of the letters “C” and “T” for 3 × 3 synaptic arrays demonstrated trainable memory behaviors using the memorizing and forgetting of STM and LTM (Fig. 6e). The letter “T” is stored as LTM, and the letter “C” is stored as STM, overwritten on one array. Measuring the current of the pixels in the array immediately after learning, it is difficult to distinguish between the two letters in the image contour clearly. However, the conductance of the letter “C” gradually decreases over time due to STM storage and eventually returns to its original state. Contrastively, the letter “T” remains stored as LTM (Fig. 6f).

### 2D TMC-Based Optoelectronic Synapse for Neuromorphic Computing

It is worth extending the controllability by an external stimulus beyond electricity, providing another degree of freedom to control the device performance and opening up new possibilities of multi-gated neural architectures [156]. In this regard, optoelectronic memristors or synapses have been proposed to detect light and store information through conductance states, which can be controlled by optical and electrical signals. 2D materials, especially 2D TMCs and their heterostructures, have great potential to be the ideal platforms for optoelectronic memristors due to their strong light-matter interactions and significant photogenerated charge trapping derived from their huge surface-to-volume ratio. Furthermore, these 2D TMCs can absorb a broad electromagnetic spectrum from ultraviolet to infrared; thus, they can be used for optoelectronic memristors and synapses for optical sensing, storage, processing [157–160]. For the efficient optoelectronic synapse operation beyond the electronic stimuli, the optical signals and gate voltages are typically used for the programming/erasing operation, and the electrical current is monitored by source–drain voltage. Both the two-terminal and three-terminal structures of optoelectronic memristors have been widely employed, mainly based on MoS_2_ and WS_2_ thin layers.

The synergy of optical and electrical stimulations leads to higher selectivity, bandwidth, and ultrafast propagation speed, facilitating the specific functional synaptic systems against external optical stimuli. As the physical properties of 2D TMC can be strongly influenced by the adjacent layers, there have been reports on optoelectronic synapses based on the heterostructure of 2D TMCs on substrates or gate dielectrics, exhibiting optical and electrical synergetic neuromorphic functions [89, 161–163]. For example, Zhou and coworkers reported a multi-functional artificial neural synapses transistor based on a hybrid heterojunction of MoS_2_/perylene-3,4,9,10-tetracarboxylic dianhydride (PTCDA) with neuromorphic functions of STP and LTP [162]. By tuning the band alignment between MoS_2_ and PTCDA, robust electrical and optical modulation modes are achieved. Compared to the artificial synapses of ion-exchange kinetics systems-based transistors, the MoS_2_/PTCDA heterojunction showed excellent synaptic plasticity due to the carrier transfer occurring at the interface of the heterojunction.

**Fig. 7:**
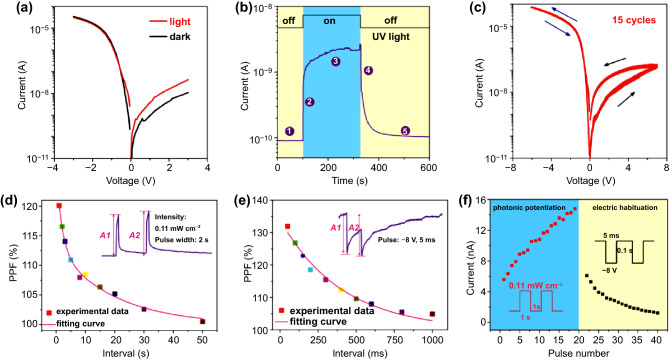
New concept of neuromorphic device application using photonic stimuli for potentiation and electric stimuli for depression via W/MoS_2_/*p*-Si vertical photodiodes. **a** photodiode behavior, **b** transient photoconductivity measurement, and **c** cyclic *I*–*V* characteristics. **d** photonic PPF properties induced by photonic pulses, **e** electric PPF properties induced by negative voltage pulses, and **f** demonstration of photonic potentiation and electric habituation with 20 cycles. Reproduced with permission from Ref. [164]. Copyright 2018, Small (Color figure online)

Guo et al. also demonstrated the ultrathin memristive synapsed based on monolayer *n*-MoS_2_/*p*-Si heterostructure to investigate the photonic potentiation and electric habituation behaviors using fabricated ultrathin memristive synapse [164]. Various synaptic functions are successfully investigated based on the inherent persistent photoconductivity performance and the volatile resistive switching behavior. The channel material, *n*-MoS_2_, was synthesized by the chemical vapor deposition (CVD) method, and the device was fabricated by transfer to *p*-Si wafer and deposition of the top electrode of W layer. In current–voltage (*I–V*) characteristics, the self-rectifying current behavior was observed with a ratio of ~ 4 × 10^3^ at a bias of 2 V under dark. Upon light illumination, the reverse-biased current is enhanced, originating from the dominated transport phenomenon through photogenerated charge carriers (Fig. 7a). Owing to the band-to-band transition, the fast response of current is observed from the photoexcitation electrons and holes. The current gradually increased over one order of magnitude because photogenerated holes get trapped and attract electrons. After turning off the light, the current rapidly decreased and showed a noticeable persistent photocurrent level (Fig. 7b). From the 15 cyclic tests, the device resistance changed from HRS to LRS, yielding hysteresis. In this device structure, the monolayer MoS_2_ plays a role in resistive switching and charge trapping/de-trapping channel (Fig. 7c). To investigate the general synaptic properties, they applied photonic pulses for potentiation and electrical pulses for habituation. A single photonic/electric pulse was given to the device for EPSC and IPSC measurement, and paired pulses were delivered to the device for verifying the PPF [152]. EPSC is triggered by two consecutive photonic pulses (0.11 mW cm^−2^, 2 s) with a time interval of 20 s between two photonic pulses. The PPF can be defined as the ratio between amplitudes of the second EPSC and the first EPSC, the PPF index. The PPF index generally is related to pulse interval, and the PPF index value gradually decreases when the time interval increases. The photogenerated carriers by the first photonic pulse are created and immobilized in the potential walls. After that, there is not enough time to recombine the photogenerated carriers with each other when the second pulses are delivered; thus, they keep being localized (Fig. 7d). Furthermore, the IPSC-induced PPF is triggered by negative electric pulses following a similar mechanism as those of EPSC-induced PPF (Fig. 7e). By utilizing the photonic stimulus-induced EPSC and electric stimulus-induced IPSC properties, the photonic potentiation and electric habituation were conducted via an optical signal with 0.11 mW cm^−2^ for 1 s and −8 V with 5 ms (Fig. 7f). The conductance of the device increases gradually due to photogenerated electrons and holes; however, electrons in MoS_2_ are driven to the interface and trapped by the trap sites in the interfacial SiO_2_ layers under the negative electric pulses [165, 166]. Finally, the conductance of the device decreased gradually with increasing electric pulse numbers, corresponding to the habituation of the synaptic strength. The MoS_2_ channel can be potentiated by electric pulses with 15 V, which is too high voltage for practical application and inefficient for low power consumption. The combination of photonic potentiation and electric habituation could be a new approach to resolve high power consumption and low device efficiency. They recently revealed that the electrical hysteresis of MoS_2_ transistors originated from the electrons trapping at the MoS_2_/SiO_2_ interfaces and the trap states come from the dangling Si–O bonds at the interfaces [167, 168]. The native SiO_2_ layer (~ 1 nm) plays a crucial role in memristive behavior. The MoS_2_ becomes less conductive when the electrons are trapped by the interfacial trap sites when the device is under the forward bias (HRS state). HRS quickly changed to an intermediate resistance state (IRS) owing to the spontaneous de-trapping of MoS_2_. The electrons are de-trapped and migrated to MoS_2_ when the device is under the reverse bias (LRS state). These experimental results are valuable for investigating the potential to investigate the potential device application of monolayer 2D TMCs to synergetic optical and electric neuromorphic functions.

**Fig. 8:**
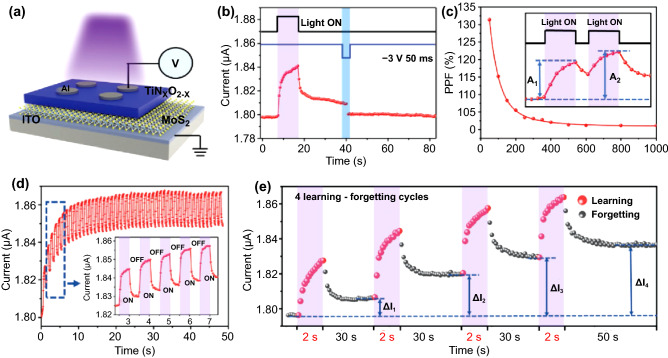
Combination of electric and photonic stimuli for demonstrating optoelectronic synaptic devices through Al/TiN_x_O_2-x_/MoS_2_/ITO heterostructures. **a** Schematic illustration of the device structure, **b** EPSC feature using photonic stimulus (3.15 mW cm^−2^, 10 s) and electrical erase with a voltage pulse (−3 V, 50 ms), and **c** light-induced EPSC-based PPF index. **d** The conductance changes under consecutive light pulses and **e** cyclic test to demonstrate the “learning–forgetting–relearning” process. Reproduced with permission from Ref. [161]. Copyright 2021, Advanced Functional Materials (Color figure online)

The emulation of human visual sense reception can be achieved through the investigation of novel optoelectronic synapses for electronic eyes and photonic neurorobotics with spontaneous response to external optical signals [169, 170]. Shen et al*.* demonstrated a heterostructured (TiN_x_O_2-x_/MoS_2_) optoelectronic synapse, exhibiting both electrical and photonic synaptic devices. They prepared ~ 50-nm-thick MoS_2_ layer on indium tin oxide (ITO) electrode by hydrothermal method and the TiN_*x*_O_2−*x*_ films grown on MoS_2_ [161]. The Al electrode is used as the top electrode. From the I–V characteristics using only voltage pulses, the conductance response versus the sweep voltage was revealed. The device conductance was gradually increased and decreased upon the consecutively positive and negative voltage sweeping. The oxygen vacancies of TiN_*x*_O_2−*x*_ could be migrated to the MoS_2_ film under the positive bias, resulting in the lower interface barrier between them and an increase of the device conductance. Reversely, the oxygen vacancies were driven back into the TiN_x_O_2-x_ films from the MoS_2_ film, leading to a decrease in conductance. In addition to the electrical signals, they demonstrated the optoelectronic synaptic behavior using 365-nm UV light with an intensity of 3.15 mW cm^−2^ (Fig. 8a). When the UV light was illuminated to the fabricated device, the conductance slightly increased during 10 s. The device current is increased abruptly because of the separation of photogenerated electron–hole pairs below the valence band. The current was slowly decayed, not rapidly recovering to the initial current level due to the inherent persistent photoconductivity effect when the light was turned off [171, 172]. These slow decay effects could be eliminated by negative voltage pulses with −5 V amplitude and 5 ms, corresponding that the device shows light-potentiation and electrical erase behavior (Fig. 8b). The PPF index induced by photonic stimuli exhibits similar characteristics compared to electrical ones, and it was decreased when the time interval between light illumination time was increased (Fig. 8c). The recombination of photogenerated excitons leads to the slower decaying current behavior and makes a big difference between electrical ones based on the back-diffusion of oxygen vacancies. Furthermore, they demonstrated that the conductance response of the fabricated devices can be controlled by light intensity and illumination time (Fig. 8d). The conductance response of the fabricated device can be controlled by light intensity and illumination time, which means the transition between short-term memory and long-term memory (STM, LTM) could be tuned by optical signals. To demonstrate the “learning–forgetting–relearning” process, they adopted the consecutive light turning on/off procedure. The conductance of the device was significantly enhanced as more photonic signals were applied (Fig. 8e). In addition to one cycle experiment, they extend their work to 4 times the learning–forgetting process to verify the reliable device operation. Each step of excitations notably potentiates the conductance levels from 4 times operation after repeating the learning or relearning processes, corresponding to the strong relationship between previously memorized information and the memory capability. Based on these findings, the advanced synaptic behaviors such as STM/LTM transition, the learning–forgetting–relearning process could be realized experimentally via the combination of photonic and electrical stimuli. Although they utilized and proposed the advanced system to emulate the human visual system, the conductance ratio and the number of conductance states are very low. Furthermore, the device shows low symmetric and nonlinear conductance behavior in LTP/LTD process, leading to low recognition accuracy. To achieve a highly efficient synaptic device using photonic/electrical combined stimuli, it is necessary to develop advanced device architecture or measurement conditions.

Alexe et al. also reported a proof-of-concept demonstration of a class of 2D optoelectronic synapses based on ferroelectric field-effect memristive transistors using a few layered WS_2_ as conducting channel and PbZr_0.2_Ti_0.8_O_3_ (PZT) thin film as the ferroelectric gate dielectric. TMDC channel [163]. By tuning the ferroelectric polarization, the WS_2_/PZT memristive transistor exhibits volatile and nonvolatile light-induced conductance switching, correlating to optically controllable short-term and long-term plasticity dynamics. It indicates that the WS_2_/PZT device can emulate complex light-controlled neuromorphic synaptic functionalities, such as short-term plasticity, long-term plasticity, and implement the optical information-driven long- and short-term memory learning rules. In addition, the authors observed the light-induced multi-level and nonvolatile conductance states of the WS_2_/PZT devices depending on the intensity, wavelength, duration, and the number of input optical pulses, indicating their high potential for developing optoelectronic synaptic with light color recognition.

### Ferroelectric 2D TMC-Based Neuromorphic Devices

From the non-centrosymmetric nature of some 2D materials, they have been initially investigated as a piezoelectric material to investigate piezoelectric or triboelectric nanogenerators for energy harvesting devices. Among them, some ferroelectric 2D TMC materials such as SnTe and α-phase In_2_Se_3_ has been successfully theoretically predicted and demonstrated to verify their near-atomic scale ferroelectricity [173, 174]. From the strong remnant polarization, delicate electrical current controllability, and the absence of depolarization effect, the 2D ferroelectrics could be one of the new functionality to overcome the current bottleneck of neuromorphic devices, which are nonlinear weight updates, low on/off ratio, a small number of conductance states, and unreliable operation. When the voltage pulses are delivered into the device, the device conductance can be modulated by controlling the degree of ferroelectric polarization [175]. Furthermore, the narrow bandgap of 2D ferroelectrics is advantageous for combining photonic and ferroelectric device integration. This section will be covered the photonic and ferroelectric neuromorphic devices based on 2D TMCs as channel materials. Various types of device configuration and optical/electrical programming/erasing methods to control the device conductance also will be introduced.

**Fig. 9:**
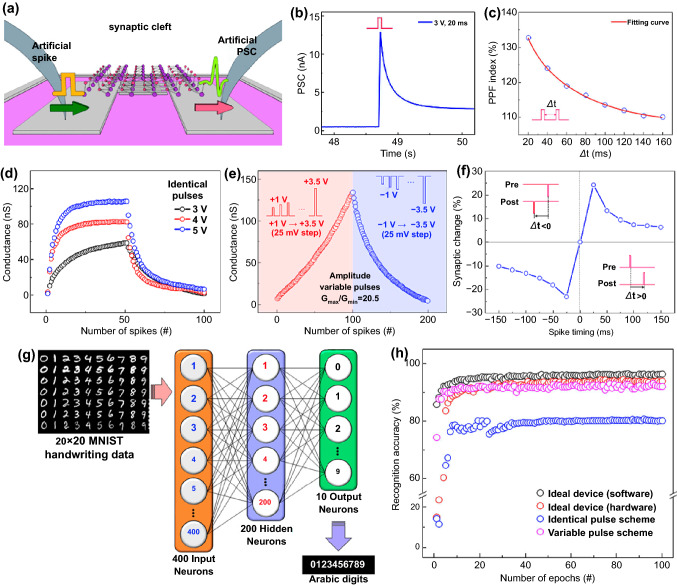
Demonstration of optoelectronic neuromorphic device application via ferroelectric α-In_2_Se_3_ thin flakes. **a** AFM image of the fabricated optoelectronic memory device composed of the α-In_2_Se_3_ channel and electrodes. **b** Schematic illustration of a proposed optical memory-based synaptic device. **c** The fatigue test with light pulses for writing and electrical pulses for erasing. **d** Retention measurement over 5000 s. The on/off ratio decreased drastically after 30 s. **e** Verifying the multi-level current switching properties using consecutive light pulses in the α-In_2_Se_3_ channel. **f** Comparison of EPSC fired by light pulses under different light intensities. **g** PPF emulation using two photonic stimuli with different light intensities and pulse intervals. **h** Demonstration of optical potentiation and electrical depression with linear relationship for 30 cycles. Reproduced with permission from Ref. [182]. Copyright 2020, Advanced Functional Materials (Color figure online)

The 2D ferroelectrics have been emerged as a new platform to achieve nanoscale ferroelectric- or ferroelectric-optoelectronic memory devices due to their robust polar ordering down to the monolayer limit, zero dangling bonds on their surfaces, and mechanical flexibility. Ferroelectric 2D TMC material (SnS) has recently emerged as one of the possible candidates for lateral device applications [176–178]. Loh et al. recently have successfully demonstrated a facile wafer-scale synthesis method for SnS thin films and its lateral metal/SnS/metal-based artificial synaptic device [179]. The SnS film was selected because of its narrow bandgap of 1.3 eV, its ability to retain room temperature ferroelectricity down to atomic scale, and its ease of scalability for large-area growth. They have investigated the in-plane ferroelectricity via various measurement methods such as second harmonic generation, piezoelectric force microscopy, and electrical switching behaviors. The ultrathin ferroelectric SnS films (3.7-nm-thick) were prepared by a chemical vapor deposition method for the wafer-scale growth. The Pt-interdigitated electrodes were adopted to apply the uniform electric field to the ferroelectric SnS thin films. The Pt/SnS/Pt device was prepared to investigate the ferroelectric current behavior and synaptic device (Fig. 9a). They demonstrated the series of neuromorphic device properties: EPSC, PPF, LTP/LTD, and STDP. By applying an electric spike of 3 V for 20 ms, the postsynaptic current (PSC) is excited and sustained over 1000 s, corresponding to the remnant polarization of SnS ferroelectric domains (Fig. 9b). The ferroelectric domains could be switched when the electrical spike is delivered over the coercive voltages, but it returned to its initial state due to insufficient electrical perturbation for permanent switching. The PPF was well demonstrated, as shown in Fig. 9c. The PPF index gradually decreased as a function of the time interval between first and second voltage spikes. Long-term plasticity is one of the critical factors which can provide a persistent increase and decrease of synaptic strength to emulate the learning mechanism of biological synapses. In Fig. 9d, the fabricated device shows good potentiation and depression behavior according to the 50 consecutive voltage spikes. When the 3 V spikes are delivered, the SnS ferroelectric domains are partially switched, and the device conductance is consistently increased and decreased by 50 potentiation/depression pulses. However, when the voltage spikes over the coercive voltage, the SnS ferroelectric domains can be completely switched; thus, the device conductance is saturated before a series of pulse spikes has been delivered. The linearity of LTP/LTD curves is significantly affected by the neural network with high learning accuracy. The linearity of the LTP/LTD curve from the identical pulse in this work is very poor because of partially switched ferroelectric domains by external stimuli. To achieve high linearity, conductance ratio, and the larger number of conductance states, they adopted the incremental voltage pulses [180, 181]. From these voltage schemes, the higher conductance states (100) with enhanced linearity (*A*_p_: −1.2, *A*_d_: −0.33) and high conductance ratio (~ 20.5) have been achieved owing to the multi-ferroelectric domains in the polycrystalline SnS thin films (Fig. 9e). They also demonstrated spike-time-dependent plasticity (STDP) learning rules which can be judged from the relationship between synaptic weight change against time interval (Fig. 9f).

**Fig. 10:**
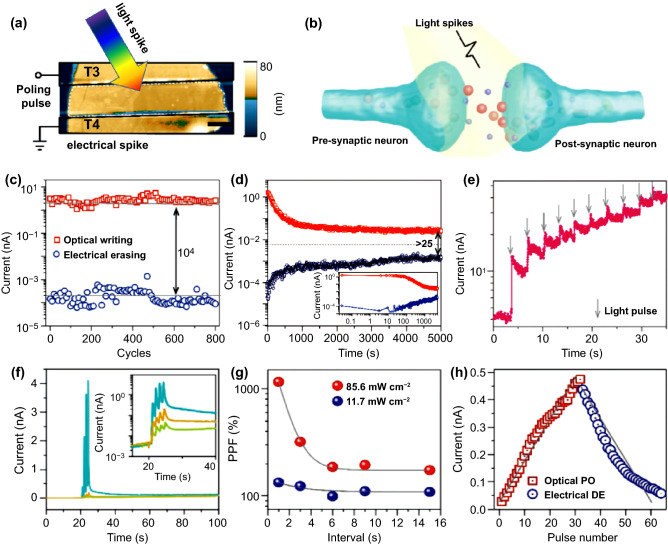
Lateral two-terminal electrical synaptic device demonstration using in-plane ferroelectric SnS thin films. **a** Illustration of fabricated Pt/SnS/Pt lateral device. **b** EPSC results triggered by 3 V and 20 ms pulse width. The partially polarized ferroelectric domains retained the current. **c** PPF emulation of the fabricated device. **d** LTP/LTD curves using identical pulses of 3, 4, and 5 V (conductance states; 50 cycles). **e** LTP/LTD curves using voltage incremental pulses (conductance states; 100 cycles). **f** STDP learning rule demonstration. **g** Artificial neural network simulation via MNIST database. **h** The recognition accuracy of i) ideal (94%), ii) identical pulses (~ 80%), and iii) variable pulses (~ 93.1%). Reproduced with permission from Ref. [179]. Copyright 2020, ACS Nano (Color figure online)

By utilizing the Modified National Institute of Standard and Technology (MNIST) handwriting database, the artificial neural network (ANN) simulation was performed to demonstrate the recognition accuracy [183]. They adopted a double-layer perceptron neural network with 400 input, 200 hidden, and 10 output neurons, as shown in Fig. 10g. From the supervised learning and training, the SnS based synaptic device achieved 92.1% accuracy after 100 training epochs compared to the 93.9% of ideal hardware-based one. The recognition accuracy is only 80.1% for the identical one, which is severely lower than the incremental one. Owing to the multi-ferroelectric domains in SnS film, it can be switched gradually by the incremental pulses so that the larger number of conductance states, the higher conductance ratio, and the better linearity can be achieved.

Another ferroelectric 2D TMC, which is in-plane and out-of-plane correlated, the α-phase In_2_Se_3_ with hexagonal and rhombohedral crystal structure has interlocked in-plane and out-of-plane dipoles at room temperature. Recently, Zhang et al. have found that the optoelectronic current behavior of hexagonal ferroelectric α-phase In_2_Se_3_ and proposed the optoelectronic nonvolatile memory device through ferroelectricity [182]. The α-phase In_2_Se_3_ flakes were prepared by the common scotch tape method, and the metal electrodes with Ti/Au (10/70 nm) were used as electrodes (Fig. 10a). They demonstrated a novel operation principle with light-induced ferroelectric domain wall motion for the new prototype of optoelectronic memory in terms of nonvolatile operation. The light-induced ferroelectric domain wall motion on α-phase In_2_Se_3_ has been investigated by piezoelectric force microscopy measurements. By sequential electrical poling and light illumination, the α-phase In_2_Se_3_ can be switchable because of their excellent photoelectric effect. These freely movable charge carriers in α-phase In_2_Se_3_ can disturb the screening effect to maintain the equilibrium between the positive intrinsic holes and negatively bounded charges, leading to the domain wall motion. From this, ferroelectric-based optoelectronic memory can be expanded to demonstrate highly efficient artificial synaptic devices. To investigate the optoelectronic memory effect, the endurance test by periodically performing optical writing and electrical erasing into the device over 800 cycles and the on/off ratio of 10^4^ can be achieved (Fig. 10c). They also observed the stable current programming/erasing current behavior until initial measurement for 30 s; however, the on/off currents gradually degraded each other, originating from the domain back switching, after 30 s (Fig. 10d). Furthermore, they monitored the dynamic current behavior as a function of the sequential light illumination for 0.8 s to identify the multi-level resistance changes. Owing to the direct and narrow bandgap of ferroelectric α-phase In_2_Se_3_, several on-current states were observed, which is intriguing for photonic synapses and photonic computing (Fig. 10e). From these results, the 2D TMC ferroelectric-based optoelectronic memories show the great potential to investigate retina-like neuromorphic vision sensors for image recognition and deep learning [62, 184, 185].

Based on these experimental results, they proposed optical power-dependent currents and multi-level current switching in α-phase In_2_Se_3_ ferroelectric-based optoelectronic memories (Fig. 10b). The two primary features, STP and LTP, have been emulated by controlling the optical power intensity. The less photogenerated carriers showed the lower distribution effect on the ferroelectric domain wall when the relatively weak light was illuminated; thus, the low on/off conductance ratio was obtained (STP emulation). By the way, the larger number of photogenerated carriers were produced by high optical power density, and they can significantly impact ferroelectric domain wall motion, showing a higher on/off ratio, corresponding to the imitation of LTP. In addition to this, by the firing of light pulse trains with different power densities, the transition process between STP and LTP can also be demonstrated (Fig. 10f). Since the PPF is one of the phenomena of the STP, it has also been emulated in this study (Fig. 10g). The optical potentiation and electrical depression were used to demonstrate the LTP/LTD curve, which is close to ideally linear weight update. This work provides a new approach to develop optical logic processing and neuromorphic vision systems using 2D TMC ferroelectric semiconductors. However, the preparation method to get large-size building blocks and low device conductance ratio should be addressed to achieve future applications.

## Challenges and Perspective

There are considerable advantages of 2D TMCs in memristor-based artificial synapses providing solutions to achieve next-generation neuromorphic computing with highly efficient logic-gated devices and large-scale chip fabrication as highlighted and discussed above. However, the demonstration of neural networks using 2D TMC has just begun and the 2D TMC based neuromorphic devices remain far from thoroughly emulating the biological synapses of the human brain. For now, the scale of the device matrix is still not large enough for computing. Although the large-area and high-density integration are essential for logic computing, a chip-level experimental verification is still lacking. For stable chip-level achievement, some technical issues need to be addressed [186].

1. *Large-scale growth of high-quality 2D TMC*: A facile large-scale growth of 2D materials is essential to integrate the circuit design. Although the high-crystalline 2D materials can be prepared by the exfoliation method, it is still challenging to prepare 2D materials with large-area and uniform physical properties. CVD is well known for a promising method to synthesize large-scale, high-quality, and uniform 2D TMCs. However, CVD-grown 2D TMCs are polycrystalline with many grain boundaries due to the limit of their single crystal growth on a micrometer scale. It is also necessary to understand how synthetic parameters (i.e., temperature, pressure, and ambient) affect the quality of 2D TMCs, such as stoichiometry, thickness, defects, and grain size because CVD-grown 2D TMCs are normally non-stoichiometric and have many defects. Therefore, the development of large-scalable, high-quality, and uniform 2D TMCs should be addressed.
2. *Modulation of physical properties and doping strategy*: Control of 2D TMCs properties is a prerequisite for designing high-performance 2D TMCs-based devices for neuromorphic computing. Doping of 2D TMCs facilitates the modulation of their electronic, optical, and structural properties. However, the conventional doping process using ion implementation is not suitable to modulate the properties of 2D TMCs due to their near-atomic thickness. To incorporate 2D TMCs into complementary circuits, a facile and stable doping process should be developed in terms of the material aspect [187]. Gas molecule-induced doping and employment of high-*k* dielectrics on 2D semiconductors would be ideal solutions to manipulate their physical properties.
3. *Low power consumption and high stability*: The energy consumption of the synaptic devices plays a critical role in the realization of neuromorphic computing with lower energy consumption. Structurally, two-terminal synaptic devices are desirable owing to their parallel, compact, and energy-efficient structure. However, the high contact resistance impedes the charge transfer in 2D TMC-based devices, leading to lower device efficiency. The deposited metal electrodes on 2D semiconductors partially damage to covalent bonding in the atomic lattice and introduce the defects that can be one of the reasons for Fermi-level pinning and forming a Schottky contact [188, 189]. Furthermore, many 2D semiconductors can be reacted or easily oxidized in the ambient condition, leading to device performance degradation. These issues can be addressed by making heterostructure with 2D electrodes (graphene, CNT, etc.) and 2D or oxide dielectrics (hBN). Although a vDW heterostructure device integration has been proposed, the feasible way to demonstrate high-density device integration has to be achieved by developing facile transfer methods and selective 2D material etching technologies. The high energy and area efficiencies are essential factors for next-generation computing. To realize beyond CMOS-based memory computing, neural network computing should be achieved with efficient power and throughput. From a reduced power consumption and novel 2D architecture, the computing system with integrated 2D materials is promising for optimizing the computation energy [190].
4. *Multi-functional synaptic devices*: In addition to electrical and optical stimuli, artificial synaptic behaviors controlled by various external stimuli, such as magnetic field, pressure, chemical adsorption/desorption, and temperature, enable the establishment of multi-gated neural architecture with sending and processing functions. By introducing a functional layer to 2D TMCs, the modulation of 2D TMC properties under external stimuli can be realized. Furthermore, integration of optical sensors and other functional devices with 2D TMC-based neuromorphic devices is challenging to develop the more advanced human-brain-inspired systems which are artificial vision and motion recognition/detection sensors.

Therefore, continuous studies should be conducted to develop many advantages of 2D materials in next-generation computing. We believe that the 2D semiconductor-based next-generation neuromorphic computing could shortly replace the current CMOS technology by addressing material preparation, facile method for device fabrication, and new design of device architectures.
