# Correction to: Memristive Devices Based on Two-Dimensional Transition Metal Chalcogenides for Neuromorphic Computing

**DOI:** 10.1007/s40820-022-00816-6

**Published:** 2022-03-05

**Authors:** Ki Chang Kwon, Ji Hyun Baek, Kootak Hong, Soo Young Kim, Ho Won Jang

**Affiliations:** 1 Department of Materials Science and Engineering, Research Institute of Advanced Materials, Seoul National University, Seoul, 08826 Republic of Korea; 2 Interdisciplinary Materials Measurement Institute, Korea Research Institute of Standards and Science (KRISS), Daejeon, 34133 Republic of Korea; 3 Department of Materials Science and Engineering, Institute of Green Manufacturing Technology, Korea University, Seoul, 02841 Republic of Korea; 4 Advanced Institute of Convergence Technology, Seoul National University, Suwon, 16229 Korea

## Correction to: Nano-Micro Lett. (2022) 14:58 10.1007/s40820-021-00784-3

The original version of this article, unfortunately, contained some mistakes and unintentional wrong description of Fig. 6 and the caption of Figs. 9, 10.

**Fig. 6:**
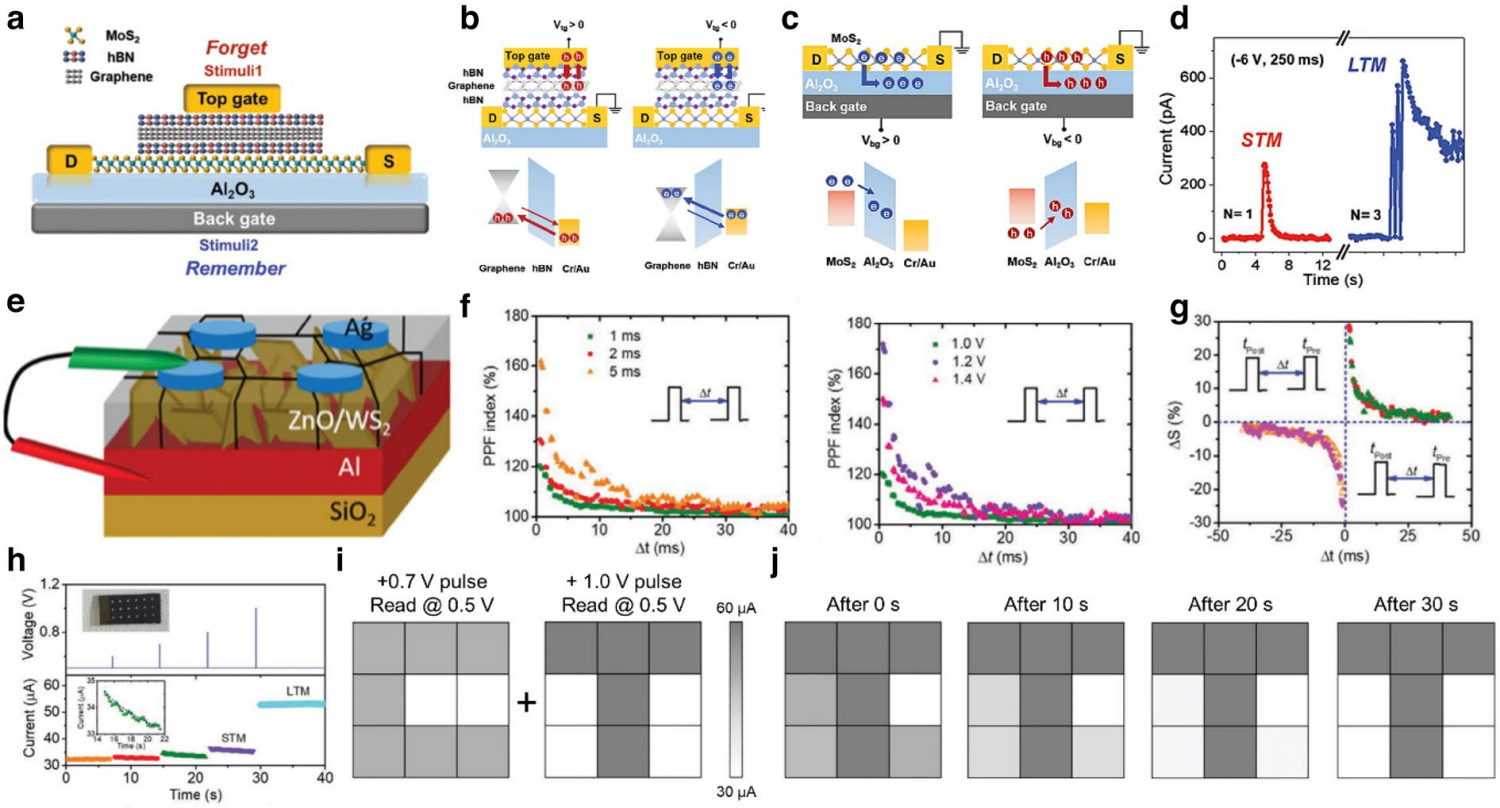
Memristor devices mediated by charge trapping and de-trapping. **a** The van der Waals heterostructure device composed of MoS_2_/hBN/graphene/hBN layers. **b** Illustration of volatile characteristics in top gate operation. **c** Illustration of nonvolatile characteristics in back gate operation. **d** Memory type transition from STM to LTM. Reproduced with permission from Ref. [116]. Copyright 2019, Advanced Science. (Color figure online) **e** Schematic structure of the Ag/ZnO/WS_2_/Al memristor. **f** PPF decays as a function of pulse interval (Δt) at different pulse amplitude and different pulse widths. **g** Experimental results for STDP. **h** The STM to LTM transition and the conductance variation by increasing input pulse voltage from + 0.7 to + 1.0 V. **i** Images of the letters “C” and “T” memorized in STM and LTM mode respectively. **j** The measured current history of the 3 × 3 arrays from right after to 30 s after the written processes. Reproduced with permission from Ref. [149]. Copyright 2019, Advanced Electronic Materials. (Color figure online)

The correct version of Fig. 6 is below. The mentioned figure name for the Fig. 6 on the manuscript should be updated.

In 2D vdW synaptic devices, the length of the tunneling barrier can be increased or decreased on the trapped or de-trapped electrons. Kumar et al. reported memristive and neuromorphic devices composed of vertically grown WS_2_ layer and ZnO (Fig. 6e) [149]. The interlayer separation between WS_2_ and ZnO layers serves as an effective porous medium allowing the ZnO to grow with defects. The interfacial region of ZnO, the very contiguous to WS_2_ layer, consists of highly non-stoichiometric oxygen-deficient condition, as confirmed by depth-dependent XPS measurements. In this defective interfacial layer, the randomly distributed oxygen vacancies play the role of charge trapping/de-trapping centers. Hence, as the programming pulse is applied, injected charge carriers fill the defects, causing internal conductance change. To realize the relevant neurological functions in synapses, various plasticities were demonstrated under diverse electric stimuli. Paired Pulse Facilitation (PPF) in neuroscience is a form of short-term plasticity manifested as strengthening the second excitatory postsynaptic potentials (EPSPs) in rapidly evoked two close spikes [151, 152]. It is shown that the PPF index decreases exponentially towards 100% as the inter-spike time *Δ*t increases from 0.1 to 40 ms, in agreement with the biological synapse (Fig. 6f). Furthermore, it can be seen that the change in the second EPSP at small *Δ*t tends to enhance with increasing spike width and voltage. Additionally, they also demonstrated the LTP, LTD, and STDP to show emulation of the synaptic device using WS_2_/ZnO heterostructure (Fig. 6g). Unlike conventional digital memory, human memory is commonly represented as short-term memory (STM) and long-term memory (LTM) [153, 154]. The STMs are lost within minutes, whereas LTMs are permanent changes, lasting from hours to years or longer [155]. In this synaptic device, a transition from STM to LTM is observed by increasing the pulse voltage from + 0.7 to + 1.0 V (Fig. 6h). Image programming of the letters "C" and "T" for 3 × 3 synaptic arrays demonstrated trainable memory behaviors using the memorizing and forgetting of STM and LTM (Fig. 6i). The letter "T" is stored as LTM and the letter "C" is stored as STM, overwritten on one array. Measuring the current of the pixels in the array immediately after learning, it is difficult to distinguish between the two letters in the image contour clearly. However, the conductance of the letter "C" gradually decreases over time due to STM storage and eventually returns to its original state. Contrastively, the letter "T" remains stored as LTM (Fig. 6j).

The corrected version of the caption for Figs. 9 and 10 is as follows:

**Fig. 9** Lateral two-terminal electrical synaptic device demonstration using in-plane ferroelectric SnS thin films. **a** Illustration of fabricated Pt/SnS/Pt lateral device. **b** EPSC results triggered by 3 V and 20 ms pulse width. The partially polarized ferroelectric domains retained the current. **c** PPF emulation of the fabricated device. **d** LTP/LTD curves using identical pulses of 3, 4, and 5 V (conductance states; 50 cycles). **e** LTP/LTD curves using voltage incremental pulses (conductance states; 100 cycles). **f** STDP learning rule demonstration. **g** Artificial neural network simulation via MNIST database. **h** The recognition accuracy of i) ideal (94%), ii) identical pulses (~ 80%), and iii) variable pulses (~ 93.1%). Reproduced with permission from Ref. [179]. Copyright 2020, ACS Nano (Color figure online).

**Fig. 10** Demonstration of optoelectronic neuromorphic device application via ferroelectric α-In_2_Se_3_ thin flakes. **a** AFM image of the fabricated optoelectronic memory device composed of the α-In_2_Se_3_ channel and electrodes. **b** Schematic illustration of a proposed optical memory-based synaptic device. **c** The fatigue test with light pulses for writing and electrical pulses for erasing. **d** Retention measurement over 5000 s. The on/off ratio decreased drastically after 30 s. **e** Verifying the multilevel current switching properties using consecutive light pulses in the α-In_2_Se_3_ channel. **f** Comparison of EPSC fired by light pulses under different light intensities. **g** PPF emulation using two photonic stimuli with different light intensities and pulse intervals. **h** Demonstration of optical potentiation and electrical depression with linear relationship for 30 cycles. Reproduced with permission from Ref. [182]. Copyright 2020, Advanced Functional Materials (Color figure online).

